# Surface‐guided tomotherapy improves positioning and reduces treatment time: A retrospective analysis of 16 835 treatment fractions

**DOI:** 10.1002/acm2.12936

**Published:** 2020-06-26

**Authors:** André Haraldsson, Sofie Ceberg, Crister Ceberg, Sven Bäck, Silke Engelholm, Per E. Engström

**Affiliations:** ^1^ Department Hematology, Oncology and Radiation Physics Skåne University Hospital Lund Sweden; ^2^ Medical Radiation Physics Department of Clinical Sciences Lund University Lund Sweden

**Keywords:** helical, radiotherapy, SGRT, surface scanning, tomotherapy

## Abstract

**Purpose:**

In this study, we have quantified the setup deviation and time gain when using fast surface scanning for daily setup/positioning with weekly megavoltage computed tomography (MVCT) and compared it to daily MVCT.

**Methods:**

A total of 16 835 treatment fractions were analyzed, treated, and positioned using our TomoTherapy HD (Accuray Inc., Madison, USA) installed with a Sentinel optical surface scanning system (C‐RAD Positioning AB, Uppsala, Sweden).

Patients were positioned using in‐room lasers, surface scanning and MVCT for the first three fractions. For the remaining fractions, in‐room laser was used for setup followed by daily surface scanning with MVCT once weekly. The three‐dimensional (3D) setup correction for surface scanning was evaluated from the registration between MVCT and the planning CT. The setup correction vector for the in‐room lasers was assessed from the surface scanning and the MVCT to planning CT registration. The imaging time was evaluated as the time from imaging start to beam‐on.

**Results:**

We analyzed 894 TomoTherapy treatment plans from 2012 to 2018. Of all the treatment fractions performed with surface scanning, 90 % of the residual errors were within 2.3 mm for CNS (N = 284), 2.9 mm for H&N (N = 254), 8.7 mm for thorax (N = 144) and 10.9 for abdomen (N = 134) patients. The difference in residual error between surface scanning and positioning with in‐room lasers was significant (*P* < 0.005) for all sites. The imaging time was assessed as total imaging time per treatment plan, modality, and treatment site and found that surface scanning significantly reduced patient on‐couch time compared to MVCT for all treatment sites (*P* < 0.005).

**Conclusions:**

The results indicate that daily surface scanning with weekly MVCT can be used with the current target margins for H&N, CNS, and thorax, with reduced imaging time.

## 1 INTRODUCTION

Accurate, reproducible, and fast setup of the patient is of great importance for a successful radiotherapy treatment, and in particular in helical tomotherapy due to the treatment complexity and number of degrees of freedom. The treatment margins are defined or calculated based on the uncertainties associated with the treatment,^1^, ^2^ and hence, affect the size of the treated volume. Helical tomotherapy^3^ is an established treatment technique where the patient is treated on a slice by slice basis using a rotating linac, megavoltage (MV) photons and a continuous couch translation. The TomoTherapy can treat targets of up to 135 cm in length in one field.^4^

Megavolt beam imaging is used for image guidance of the patient setup.^5^ The treatment beam is combined with an on‐board single row computed tomography (CT) detector array and the captured projection images are used to reconstruct a volumetric MVCT image of the patient.^6^ Daily imaging using MVCT contributes to absorbed dose outside the treatment volume.^6^ Also, MVCT is time consuming which decrease the patient throughput, and contributes to an increased risk of intra‐fraction patient movement.^7^ To reduce the amount of MVCT images while keeping an accurate patient setup several imaging strategies have been adopted, such as weekly MVCT imaging with daily patient setup using in‐room lasers.^8^ A recent strategy is to use surface guided radiotherapy (SGRT), where the patien'ts skin surface is scanned by an optical surface scanning (OS) system for patient setup.^9^ The OS system compare the patient's surface at treatment setup to a reference surface and accurately calculates the patient position.^9^ The advantage of using surface scanning is that the information from the surface can improve the patient setup compared to in‐room lasers.^10^, ^11^, ^12^ Also, a surface scan takes seconds, in comparison to minutes for MVCT. Thus, surface scanning has the potential to increase the accuracy, without substantially adding time for setup compared to setup with in‐room lasers. The surface can be correlated to the MVCT images with a similar method as the in‐room lasers. In this study, the Sentinel surface scanning system (C‐Rad, Uppsala, Sweden)^9^ was used to position the patients at a TomoTherapy HD (Accuray, Madison, US) linac between 2012 to 2018. Crop et al has previously reported improved patient setup for breast cancer patients using SGRT at tomotherapy^12^; however, in this study an extensive number of targets in head and neck (H&N), intra‐ and extracranial (CNS), thorax and abdomen were included. The aim of this study was to retrospectively investigate the potential improvements of surface guided setup compared to in‐room lasers, both verified by weekly MVCT. Also, the potential time gain using SGRT compared to daily and weekly MVCT was to our knowledge investigated for the first time.

## 2 MATERIALS AND METHODS

### 2.A Positioning

#### 2.A.1 Surface scanning

The Sentinel OS system is a laser‐based OS system that acquires a three‐dimensional (3D) surface image of the patient over several seconds. The daily surface scanned is registered to a reference surface and the patient’s position is calculated using rigid registration.^9^ The scanner is mounted in the ceiling, at the end of the treatment couch. To avoid shadowing of the surface due to the closed bore of the TomoTherapy, the patient setup was carried out at the virtual isocenter, 700 mm longitudinal outside the bore. The Sentinel OS system has been found to be reproducible to < 1 mm and < 1° of rotation.^13^ The Sentinel system and the TomoTherapy lack communication, and thus for safety any couch shifts that were carried out based on the OS system was followed by a second surface scan to verify that the shifts were carried out correctly.

#### 2.A.2 Megavoltage computed tomography

The standard imaging modality on the TomoTherapy is MVCT acquired using a built‐in detector array with the treatment beam at 3.5 MV energy. The collimator is positioned in the longitudinal direction and was set to 4 mm width for imaging. Images were acquired slice‐by‐slice and using a pitch set to fine, normal, or coarse. The reconstruction interval was 2 or 4 mm optionally. Transversal slice spatial image resolution for MVCT imaging was ≤ 1.6 mm per pixel at 512 × 512 pixels. The scan length for MVCT imaging was chosen to include the PTV in the longitudinal direction. The MVCT image was reconstructed and compared to the reference CT using automatic registration with manual adjustment. The patient was repositioned if the automatic registration resulted in a rotation of more than 2°. If the patient was repositioned, a second scan was acquired. The registration was further performed with only translational axis, the correction was applied, and the couch was moved from the control room. Prior to 2012, the couch was controlled solely from inside the treatment room, which increased the setup and imaging time.

#### 2.A.3 Positioning procedure

**Fig. 1:**
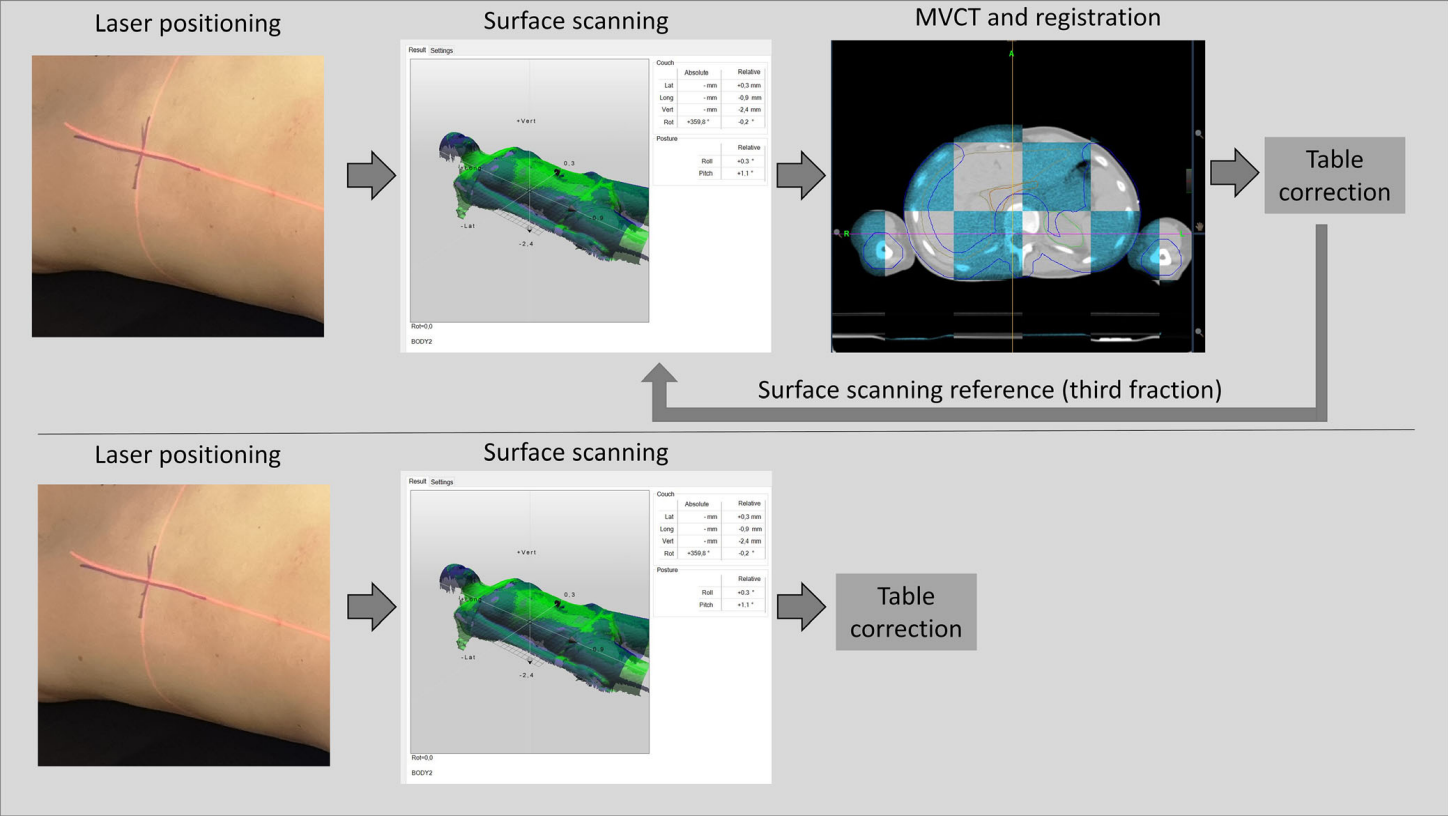
[Daily workflow for surface scanning positioning procedure]. The first three fractions laser based setup was followed by surface scanning and then megavoltage computed tomography (MVCT) imaging. After table correction on the third fraction a new surface scanning reference was acquired provided that the surface scan based correction and the MVCT based correction correlated (top). The following fraction MVCT imaging was omitted and the table correction was based on surface scanning (bottom). The surface scanning was checked with weekly MVCT imaging

Prior to CT, patients were immobilized with either a thermoplastic mask (Orfit Industries, Wijnegem, Belgium), a vacuum bag (VacFix, Par Scientific A/S, Odense Denmark), or a light mattress. Head and neck patients were immobilized using a 5‐points mask, CNS patients using a three‐point mask, and thorax and abdomen patients using either a light mattress or vacuum bag. The patients were positioned in three steps; (a) with in‐room lasers with external markers as reference, (b) with surface scanning matched to a reference surface, and (c) using MVCT with the planning CT as reference. This procedure was performed at the first three fractions. On the third fraction, after MVCT couch correction, a surface scan was acquired to use as reference surface during the following fractions. For ensuing treatment fractions the patients were positioned daily with first in‐room lasers followed by surface scanning, and weekly MVCT for verification of positioning and internal anatomy. The weekly MVCT imaging was performed after in‐room laser and surface scanning setup correction. Thus, each patient was positioned by laser, followed by surface scanning and MVCT for three fractions. A surface scan reference based on MVCT and couch correction performed on the third fraction was then used as primary position procedure except for MVCT scans performed once weekly, Fig. 1. The procedure has been derived from the work of Månsson,^14^ which concluded that weekly imaging with laser setup and three initial imaging verification procedures were sufficient with the used imaging protocol. The threshold for deviation between MVCT and surface scanning was 2 mm in any direction. A deviation larger than 2 mm prompted MVCT the following treatment fraction, as did any large anatomical changes. The protocol at the time of the study was a NAL protocol^8^ with action limits of 2 mm for H&N and CNS, for thorax and abdomen patients the action limit was 3 mm. CTV to PTV margins differ between sites and diagnosis, but was generally 5–7 mm for CNS and H&N and 7–10 mm for thorax and abdomen patients.

#### 2.A.4 Positioning data statistics

**Fig. 2:**
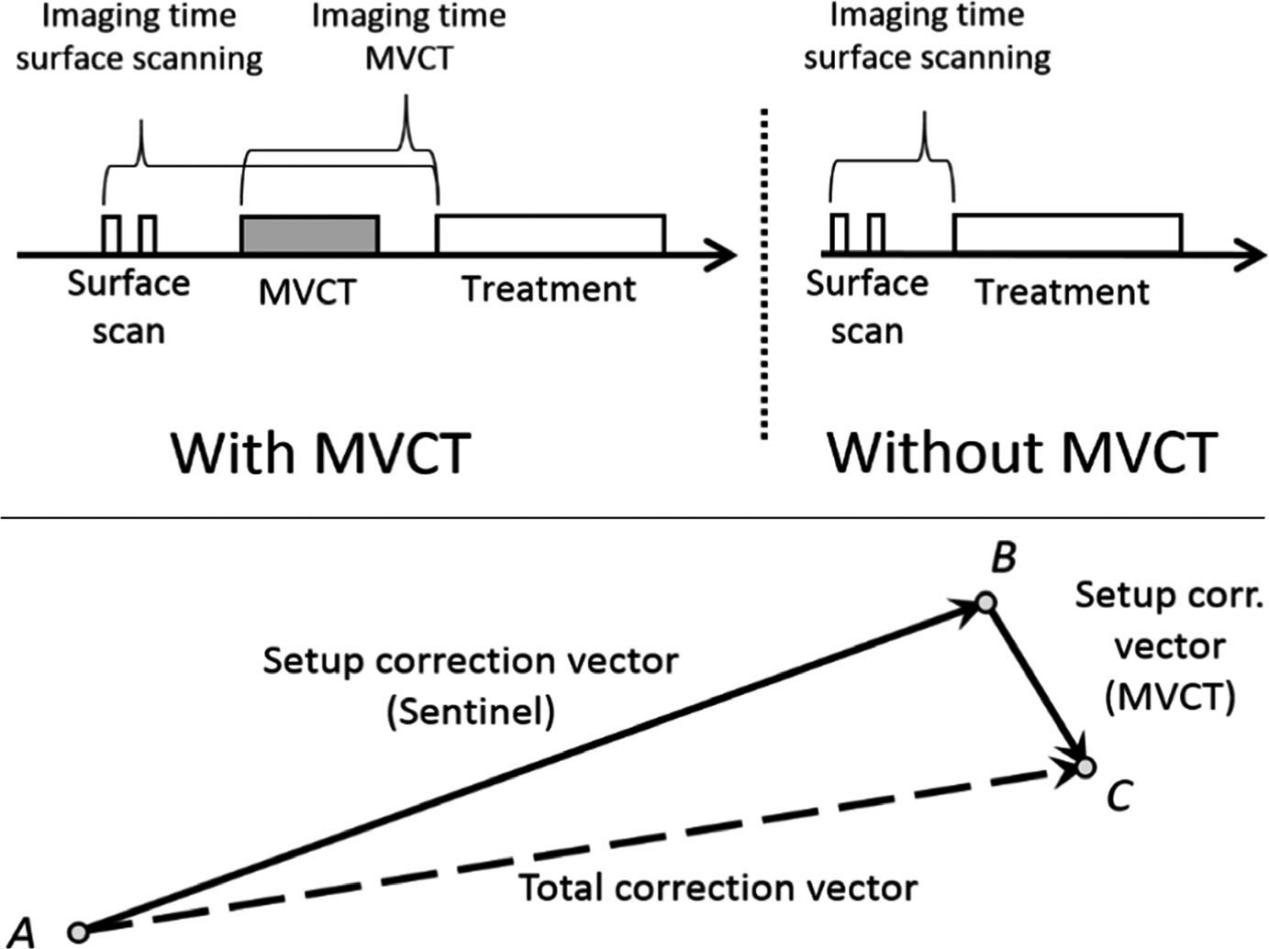
[Setup correction vector and imaging time]. For surface scanning the time includes any following surface scans, couch movement, repositioning of the patient and megavoltage computed tomography (MVCT) imaging up till beam on. MVCT was performed for first three fractions and weekly if no relevant anatomical deviation was found and if the difference between MVCT and surface scanning was < 2 mm in any direction. The definition of the total setup correction vector (bottom) is here visualized as a sum of the individual correction vectors

The MVCT was registered with the planning CT as reference. The resulting translational couch movement from the original position to the registered position was defined as the *setup correction vector,* Fig. 2. The positional data was quantified by randomly selecting one MVCT image setup correction vector per plan. Random selection was used to avoid overestimation of the confidence interval, since the treatment fractions is correlated to the patient, we cannot simply sum all treated fractions for all patients without any correction. In addition, the number of fractions varied between patients. This correction vector was used to assess the residual setup deviation of the surface scanning performed prior to the MVCT. One setup correction vector from the surface scanning was selected at random to assess the residual setup deviation between in‐room lasers and surface scanning. The correction vector from the surface scanning was added to the MVCT correction vector to measure the total residual error between the in‐room laser setup and the registered based on the MVCT image. The setup data from the first three fractions were omitted from the analysis since the reference surface was acquired during the third fraction. In addition, correction for systematic deviations was simulated by calculating a correction factor based on the first three fractions adapted from de Boer et al. and Bortfelt et al.,^8^, ^15^

(1) $$\begin{array}{c} c_{p}=-\frac{N}{N+1}\sum_{i=1}^{N}\frac{\underset{X_{i}}{\to }}{N} \end{array}$$

where *c_p_* was the setup correction for patient *p* that was corrected for the *N* first fractions with the setup correction vector $\vec{x_{i}}$ . The correction factor was applied to the remaining fractions thus simulating a systematic correction. The Mann‐Whitney U test was used for hypotheses testing.

### 2.B Imaging time

The time from imaging start to beam‐on was defined as the *imaging time*, Fig. 2. The imaging time for surface scanning was calculated from the first surface scan to beam‐on and thus included any following surface scans, MVCT procedures, registration, and couch translations. Similarly, the imaging time for MVCT was defined from the first MVCT scan to beam‐on, including any following scans and registration or realignment of the patient. The imaging time for one fraction was randomly selected per treatment plan and imaging modality. The difference in total imaging time per fraction was tested against the null hypotheses using the Mann‐Whitney U test, since normality could not be assumed. The imaging time was then multiplied by the number of treatment fractions to yield the total time difference per treatment plan.

### 2.C Data selection

The data were collected between January and April 2018. Data were gathered retrospectively as all patients treated with TomoTherapy from the time period 2012–2018 and included treatments to the head and neck, CNS, thorax, and abdomen or pelvic area. Patients that received treatment to the abdomen and pelvis were included in the same treatment site group called abdomen. Only patients with more than three treatment fractions were included and patients positioned using in‐room lasers, surface scanning, and MVCT performed on more than three fractions. Data was extracted from the Sentinel database and from the TomoTherapy archive using an in‐house developed C# program. The resulting data were analyzed using Python (Version 3.6, Python software foundation, 2019).

## 3 RESULTS

A total of 696 patients with 894 plans were analyzed – in total 16 835 treatment fractions. Of the 894 plans, 78 plans were undefined treatment sites or treatment sites other than H&N, CNS, thorax, or abdomen and thus omitted from the analysis.

### 3.A Positioning

**Fig. 3:**
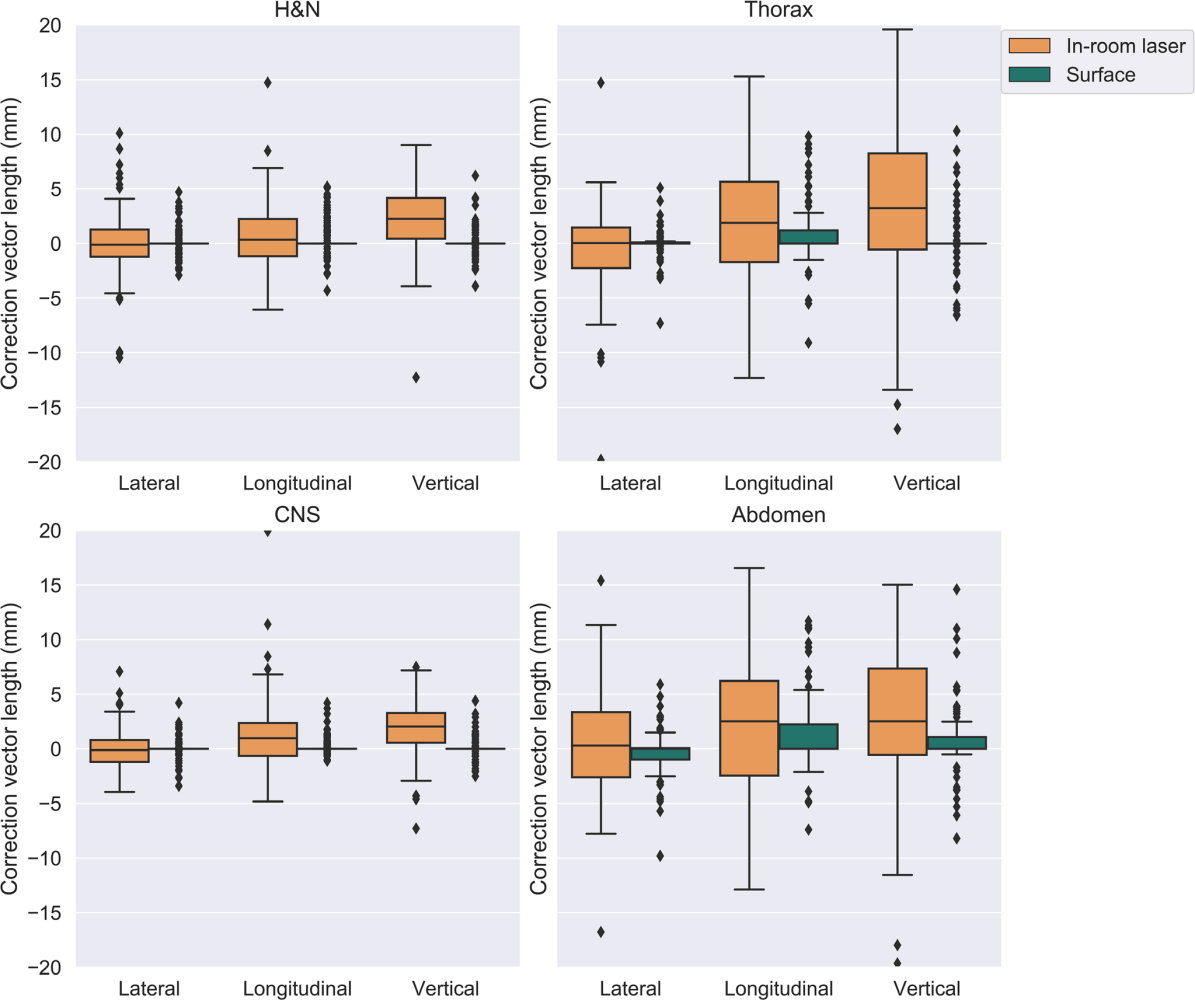
[Setup correction per axis and image modality] The residual error for surface scanning and the residual error for in‐room lasers, plotted per axis and treatment site. The residual error was assessed from the setup correction with megavoltage computed tomography to CT. Shown as a box‐and‐whisker plot, where the mid‐line represents the median (line), the interquartile range (box) and 1.5 times past the quartile range (outer line) and outliers (black point)

**Fig. 4:**
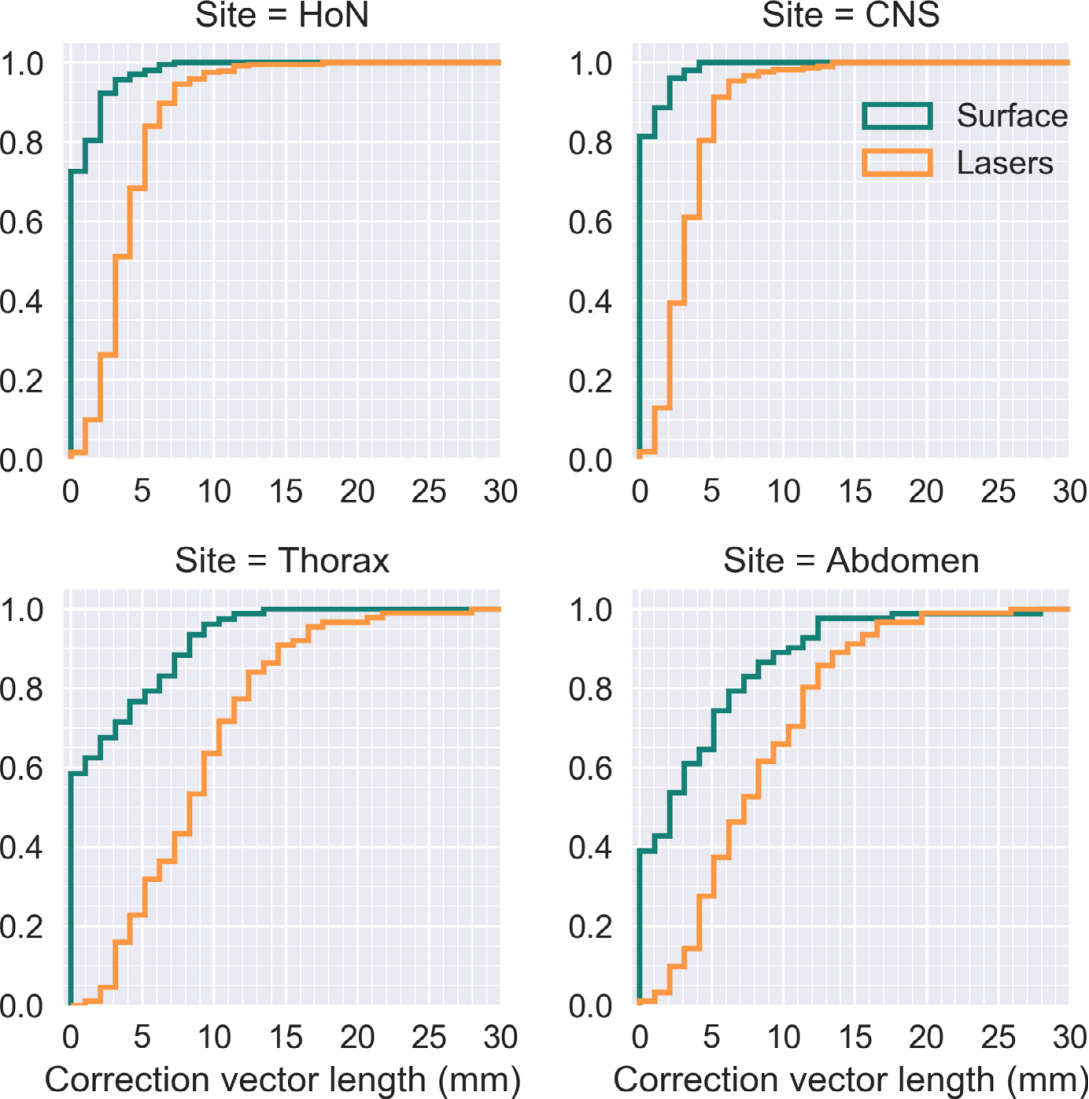
[Length of setup deviation per image modality] residual error for in‐room lasers and surface scanning as assessed by the sum of the megavoltage computed tomography (MVCT) and Sentinel correction vector (orange) and the MVCT setup correction vector (green) respectively. Here plotted as the cumulative sum of the setup correction deviation

For patients immobilized with 3‐ or 5‐point mask (CNS and H&N), only 1.7% of the fractions positioned with surface scanning had a residual error larger than 5 mm, compared to laser‐based setup where 27.5% of the fractions had a residual error larger than 5 mm. When in‐room lasers are corrected for systematic error based on the first three fractions, 11.8% of the fractions had a residual error larger than 5 mm. The difference in length of the residual error between in‐room lasers and optical surface scanning was significant (*P* < 0.005) for all sites. We compared the residual error to assess the positioning accuracy of in‐room lasers and optical surface scanning (Figs. 3 and 4), as well as residual error per axis, Table 1. The smallest residual errors are seen for cranial and head and neck patients, with larger setup residual errors for the thorax and abdomen treatment sites, Figs. 3 and 4. On average, the difference between the residual error per axis was 1.7, 2.9, and 2.5 mm for the lateral, longitudinal and the vertical axis respectively. For in‐room lasers, the largest error was mostly found in the vertical direction followed by the longitudinal direction. For surface scanning, the largest error was mostly in the longitudinal direction followed by the lateral direction. The residual error was further separated into a systematic and a random error,^16^ Table 2. The random and systematic error was substantially larger for in‐room lasers than optical surface scanning, for all sites. The number of MVCT scans that prompted a rescan due to the difference between surface scanning and MVCT was over the action limit was for H&N.

**Table 1:** [Residual setup error] Residual setup error for in‐room lasers and surface scanning as assessed by the megavoltage computed tomography correction, per treatment site. The 50% and 90% percentile are tabulated over the different axis together with the length of the error vector. Two millimeters at the 90% percentile is interpreted as 10% of all values are over 2 mm. The setup vectors were randomly selected, one per patient. lat = lateral couch direction, long = longitudinal couch direction, vrt = vertical couch direction. Error length is the length of the image correction vector against the reference image, that is, the residual positioning deviation after surface scanning and in‐room laser positioning respectively

Site	Percentile (%)	Surface scanning				In‐room lasers			
		x (mm)	y (mm)	z (mm)	Length (mm)	x (mm)	y (mm)	z (mm)	Length (mm)
HoN	50	0.0	0.0	0.0	0.0	1.1	1.6	2.7	4.0
CNS	50	0.0	0.0	0.0	0.0	1.2	2.0	2.5	3.6
Thorax	50	0.0	0.0	0.0	0.0	2.5	3.6	6.2	8.4
Abdomen	50	0.7	1.0	0.5	2.6	3.3	4.2	6.0	8.8
HoN	90	1.5	2.1	1.4	2.9	4.9	4.5	4.5	8.1
CNS	90	0.9	0.6	1.2	2.3	2.3	4.2	4.0	6.3
Thorax	90	2.0	5.7	5.9	8.7	5.3	7.2	14.9	15.7
Abdomen	90	3.3	7.4	5.4	10.9	5.0	9.8	12.5	17.5

**Table 2:** [Deviations from megavoltage computed tomography imaging for surface scanning and in‐room lasers] Systematic and random residual error based on the setup for in‐room lasers and surface scanning respectively, tabulated with simulated correction for systematic error based on the first three fractions for in‐room laser positioning (Laser NAL). Calculated from all treated setup vectors (N = 16 835). All values are presented in mm

Site	Axis	Systematic			Random	
		Laser	Laser NAL	Surface	Laser	Surface
H&N	Lateral	1.3	0.7	0.4	2.0	0.7
	Longitudinal	1.6	1.6	0.8	2.4	1.2
	Vertical	2.6	1.3	0.4	2.6	2.1
CNS	Lateral	1.0	0.6	0.2	0.8	0.5
	Longitudinal	1.4	1.0	0.3	1.7	0.6
	Vertical	2.1	1.2	0.3	2.7	0.6
Thorax	Lateral	2.2	1.4	1.2	2.8	1.4
	Longitudinal	3.6	3.2	3.2	5.9	4.0
	Vertical	5.2	2.5	1.9	5.0	2.5
Abdomen	Lateral	2.3	1.9	1.6	2.6	1.1
	Longitudinal	3.5	2.9	3.1	5.6	3.3
	Vertical	5.0	3.5	2.9	5.7	2.5

### 3.B Imaging time

**Fig. 5:**
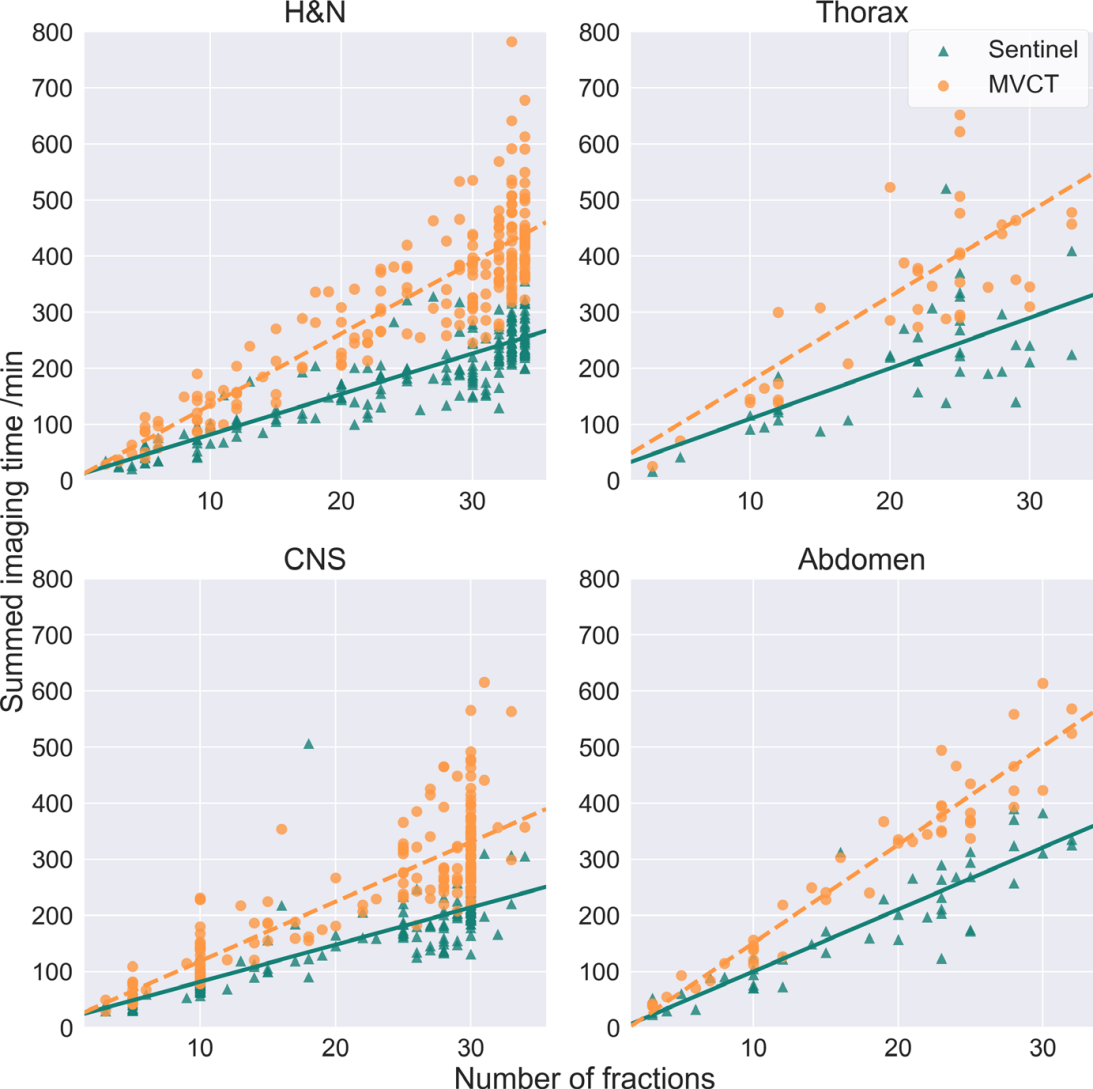
[Total imaging time per modality] Accumulated imaging time against number of fractions per treatment plan, for imaging with Sentinel and with megavoltage computed tomography (MVCT) respectively. For surface scanning with sentinel, the time from first imaging to beam‐on for each fraction was summed per treatment plan which includes all MVCT scans taken weekly. For MVCT, the time from first imaging to beam‐on for each fraction with MVCT imaging, was divided with the number of MVCT imaging procedures per plan and multiplied with the number of fractions to simulate daily imaging with MVCT for comparison

The difference in imaging time was assessed as total imaging time per treatment plan, modality, and treatment site, Fig. 5, where total imaging time per treatment plan refers to the accumulated time for each image modality as in all the weekly MVCT and surface scans for the length of the treatment for that plan. For the image modality MVCT that refers to the accumulated MVCT imaging time for the entire treatment as if the MVCT was taken daily. Patients that received treatment prior to the upgrade of the couch movement from the control room was excluded from the time analysis. The difference in total imaging time between surface scanning and MVCT per fraction was significant for all sites (*P* < 0.005). The mean time saved per fraction for a head and neck patient receiving 34 fractions was 4.8 min (σ = 0.8 min) and for a CNS patient with 30 fractions the mean saved time per patient was 3.7 min (σ = 0.5 min) when positioning with surface scanning. Similar mean saved time was achieved, 4.0 min (σ = 0.9 min) and 3.4 min (σ = 0.7 min) for patients receiving treatment to the thorax and abdomen with 34 and 30 fractions respectively.

## 4 DISCUSSION

In this study, we analyzed a total of 16 835 treatment fractions over a 7‐yr period. The large dataset enables analysis of subgroups and contributes to robust statistics. However, the following uncertainties have been identified in the study; the treatment machine was changed from Hi‐Art to HD in 2012 and several upgrades has been made during this period. In addition, the possibility to move the couch from the control room was installed in 2013, which may add uncertainties to the data. Prior to analysis, data with implausible values were removed.

With in‐room laser based setup, the largest magnitude of setup deviation was in the vertical direction, which is consistent with other studies.^17^, ^18^, ^19^ This deviation was found to be systematic for all treatment sites, and in the positive direction meaning that the couch is generally too low when compared with MVCT imaging. The effect stems from the positioning being performed in the virtual isocenter, 700 mm outside the bore. When the table top is moved into treatment position inside the bore, the couch sags, usually 1–3 mm. This effect has been reported in other studies.^17^, ^18^ The error in the vertical direction was almost entirely compensated with the surface scanning since the surface reference was acquired after MVCT imaging and setup correction and hence corrected for the sag, as the MVCT imaging is done inside the bore/in treatment position. This type of systematic deviation can be compensated with in‐room laser setup, if the setup deviation is larger than the tolerance.

The image pitch was set to fine for patients immobilized with thermoplastic mask, and normal or fine for other patients in the study. The reconstruction interval normally used in each case was 2 or 4 mm. The resolution has been shown to affect to possible registration accuracy as compared to an independent system,^20^ but the deviations were in general in the submillimeter range and at least half that of the voxel resolution for the investigated phantom.

The largest setup deviation with surface scanning was found in the longitudinal direction. At our clinic, the Sentinel was mounted in the ceiling at the foot end of the couch. This allows space during service to remove the covers of the TomoTherapy. The downside is the shallow angle to the patient. This problem could be managed by mounting the camera on a rail closer to the bore, allowing the camera to be moved during service of the TomoTherapy. This mounting can increase the uncertainty in camera mounting position and increases the QA workload. To better solve the uncertainty in longitudinal positioning with surface scanning, we are currently placing Styrofoam cubes on the patient to better aim the surface scanning for abdomen and thoracic patients. In this study, the effect of rotational deviations has not been tested. If the Sentinel or the registration of MVCT to CT indicated any rotation outside tolerance, the patient was readjusted in their immobilization.

There was a notable difference between patients immobilized with mask and other fixations. This can be attributed to the immobilization but also to the distance from surface to target, which is generally greater for thoracic and abdomen patients. In addition, thoracic patients and abdomen patients may exhibit larger intra‐ and inter‐fractional movement of target relative to surface. Scanning of the CNS and H&N patients is mainly based on the mask, but the scanning for positioning seems to work well in most cases, as seen in the results. Problems such as weight loss and rotation inside the mask is hard to spot under the mask fixation, this is why using open masks can be an alternative when using surface scanning for setup. To account for any large anatomical changes, the treatment personnel was trained to monitor the response on the surface scanner in areas commonly associated with weight loss, such as the abdomen, large changes prompted MVCT rescans and acquiring of a new reference surface. An added benefit of the surface scanning compared to MVCT is the potential larger field of view, positioning of arms and shoulders can be better imaged with surface scanning. This is especially important with total marrow and total skin irradiations which have large target areas, extending wider than the field of view. Regarding abdomen and thoracic patients, we believe that surface scanning can be suitable for certain subgroups where the surface and target does not exhibit any large intra‐ or inter fractional movement in relation to each other. Further analysis within the subgroup would be needed to clarify which subgroups is suitable for surface scanning.

We found that with surface scanning only 1.7% of the setup deviations was larger than 5 mm for H&N and CNS, which was the target margin for patients immobilized with a thermoplastic mask. To the authors knowledge, this is the only setup protocol to achieve this accuracy apart from daily imaging with MVCT. Other studies have investigated the residual errors for different treatment sites with daily in‐room lasers,^18^, ^21^, ^22^, ^23^, ^24^ using no action limit protocols (NAL)^8^ and determined the residual deviation after daily in‐room and NAL to be 2.6–14.2% for head and neck patients, depending on the number of fractions for evaluation and action limit. This would imply that a protocol with weekly MVCT imaging using daily surface scanning is as good or better than setup with in‐room lasers and NAL protocol. Our positioning data was found similar to published data with an older laser scanning system.^19^ To improve the positioning with surface scanning, a NAL protocol could be implemented based on the first three fractions, or by evaluating similar to methods on conventional linac.^8^ Despite the high accuracy, there will be a few fractions that will be outside the treatment margins. Similar to population based margin recipes were the margins is deducted were 90% of the population receives 95% of the prescribed dose a imaging protocol which does not include daily imaging should prompt a discussion on each clinic if the treatment margins are sufficient and what are the effects depending on the fractionation.

We found the difference in imaging time between daily surface scanning and daily MVCT significant (*P* < 0.005). The average time saved was reasonable considering the imaging and registration time for the different patient groups. A possible source of uncertainty was that beam‐on as saved in the archive is the press of the beam‐on button which can differ from the actual beam on.

It has been shown that a shorter treatment time can decrease the positioning uncertainty,^12^ since patients treated for radiotherapy exhibit a baseline drift during treatment that is time dependent,^7^, ^25^, ^26^, ^27^, ^28^, ^29^ and this shift has a dosimetric impact^30^ on critical structures. We compared daily MVCT imaging to the use of three initial MVCT imaging followed by weekly MVCT imaging, with surface scanning as setup tool on fractions without MVCT. This potential reduces the number of scans from 34 to 9 for a normal head and neck patient, not only reducing the time for acquiring the image but also the registration time. The time required for adjustments based on the surface scanning is in part negated by the assistance it provides to position the patient on the couch For the time saving to have effect on throughput, any time saved must be adjusted in the time slot in the booking system and to fill those slots. The actual throughput effect of surface scanning has in that regard not been tested. There has been few publications discussing patient throughput on Tomotherapy, but our results are consistent with literature where an increased throughput of 40% has been observed where surface scanning has been used instead of MVCT imaging for H&N,^31^ and when surface scanning was used for total marrow irradiation a time saving of 25 min was seen on average.^32^

The decrease in the number of MVCT scans could also potentially save normal tissue from imaging dose. The population effects need to be further analyzed, but the dose from one MVCT image is typical in the range of 2–3 cGy^33^ which would imply a dose saving of approximately 60 cGy for a treatment with 34 fractions, if weekly MVCT and SGRT is compared to daily MVCT.

The result can be used to save time at the linac compared to daily MVCT or shrink the target margins compared to daily setup with in‐room lasers. This has the potential to save dose to normal tissue and to increase throughput at the treatment machine. The actual implications of the setup deviations on the PTV margin should be further investigated. In addition, how the time reduction for patient on couch affects the intra‐fraction motion and how NAL and surface scanning in TomoTherapy can be combined are areas of interest for further research.

## 5 CONCLUSIONS

Optical surface scanning based setup on TomoTherapy has significant lower setup error as compared to in‐room lasers based setup for all site, H&N, CNS, thorax and abdomen. Surface scanning was found to result in low setup error compared to the target margins for all sites but abdomen. In addition, surface scanning with weekly MVCT was found to significantly reduce the average patient on‐couch time compared to daily MVCT. The results indicate that daily surface scanning with weekly MVCT can be used with the current target margins for H&N and CNS. The largest gain for surface scanning was found with H&N which had large difference in deviation from MVCT as compared to lasers, and the group also had a large time gain when the number of MVCT scans were reduced. The setup deviation was large for thoracic and abdomen patients, but further analysis is needed for those subgroups to assert if they are suitable for surface scanning.

## CONFLICT OF INTEREST

The authors have no relevant conflict of interest.

## References

[1] Guckenberger M , Meyer J , Vordermark D , Baier K , Wilbert J , Flentje M . Magnitude and clinical relevance of translational and rotational patient setup errors: a cone‐beam CT study. Int J Radiat Oncol Biol Phys. 2006;65:934–942. 16751076 10.1016/j.ijrobp.2006.02.019

[2] Landberg T , Chavaudra J , Dobbs J , et al Report 50. J Int Comm Radiat Units Meas. 1993;os26:NP.

[3] Mackie TR , Holmes T , Swerdloff S et al Tomotherapy: a new concept for the delivery of dynamic conformal radiotherapy. Med Phys. 1993;20:1709–1719. 8309444 10.1118/1.596958

[4] Accuray I . TOMOTHERAPY® H™ SERIES: TomoH™, TomoHD™ and TomoHDA™ Systems Technical Specifications. In: Accuray I, ed. Madison, WI, US; 2017.

[5] Mackie TR , Kapatoes J , Ruchala K , et al Image guidance for precise conformal radiotherapy. Int J Radiat Oncol Biol Phys. 2003;56:89–105. 12694827 10.1016/s0360-3016(03)00090-7

[6] Ding GX , Alaei P , Curran B , et al Image guidance doses delivered during radiotherapy: quantification, management, and reduction: report of the AAPM Therapy Physics Committee Task Group 180. Med Phys. 2018;45:e84–e99. 29468678 10.1002/mp.12824

[7] Jensen CA , Acosta Roa AM , Lund JA , Frengen J . Intrafractional baseline drift during free breathing breast cancer radiation therapy. Acta Oncol. 2017;56:867–873. 28464748 10.1080/0284186X.2017.1288924

[8] de Boer HC , Heijmen BJ . A protocol for the reduction of systematic patient setup errors with minimal portal imaging workload. Int J Radiat Oncol Biol Phys. 2001;50:1350–1365. 11483348 10.1016/s0360-3016(01)01624-8

[9] Brahme A , Nyman P , Skatt B . 4D laser camera for accurate patient positioning, collision avoidance, image fusion and adaptive approaches during diagnostic and therapeutic procedures. Med Phys. 2008;35:1670–1681. 18561642 10.1118/1.2889720

[10] Stanley DN , McConnell KA , Kirby N , Gutierrez AN , Papanikolaou N , Rasmussen K . Comparison of initial patient setup accuracy between surface imaging and three point localization: a retrospective analysis. J Appl Clin Med Phys/Am Coll Med Phys. 2017;18:58–61. 10.1002/acm2.12183 PMC5689923 28901684

[11] Kugele M , Mannerberg A , Norring Bekke S , et al Surface guided radiotherapy (SGRT) improves breast cancer patient setup accuracy. J Appl Clin Med Phys/Am Coll Med Phys. 2019;20:61–68. 10.1002/acm2.12700 PMC6753725 31478615

[12] Crop F , Pasquier D , Baczkiewic A , et al Surface imaging, laser positioning or volumetric imaging for breast cancer with nodal involvement treated by helical TomoTherapy. J Appl Clin Med Phys/Am Coll Med Phys. 2016;17:200–211. 10.1120/jacmp.v17i5.6041 PMC5874112 27685103

[13] Pallotta S , Marrazzo L , Ceroti M , Silli P , Bucciolini M . A phantom evaluation of Sentinel(), a commercial laser/camera surface imaging system for patient setup verification in radiotherapy. Med Phys. 2012;39:706–712. 22320780 10.1118/1.3675973

[14] Månsson S . Patient Positioning Correction Strategies in Radiotherapy: A Portal Imaging Study. Lund: Lund University; 2004 https://www.lunduniversity.lu.se/current‐students/academic‐matters‐support/lup‐student‐papers

[15] Bortfeld T , Mv H , Jiang SB . When should systematic patient positioning errors in radiotherapy be corrected? Phys Med Biol. 2002;47:N297–N302. 12502052 10.1088/0031-9155/47/23/401

[16] Yan D , Wong J , Vicini F , et al Adaptive modification of treatment planning to minimize the deleterious effects of treatment setup errors. Int J Radiat Oncol Biol Phys. 1997;38:197–206. 9212024 10.1016/s0360-3016(97)00229-0

[17] Schubert LK , Westerly DC , Tome WA , et al A comprehensive assessment by tumor site of patient setup using daily MVCT imaging from more than 3,800 helical tomotherapy treatments. Int J Radiat Oncol Biol Phys. 2009;73:1260–1269. 19251098 10.1016/j.ijrobp.2008.11.054 PMC2749998

[18] Saha A , Mallick I , Das P , Shrimali RK , Achari R , Chatterjee S . Evaluating the need for daily image guidance in head and neck cancers treated with helical tomotherapy: a retrospective analysis of a large number of daily imaging‐based corrections. Clin Oncol (R Coll Radiol). 2016;28:178–184. 26746002 10.1016/j.clon.2015.11.014

[19] Moser T , Habl G , Uhl M , et al Clinical evaluation of a laser surface scanning system in 120 patients for improving daily setup accuracy in fractionated radiation therapy. Int J Radiat Oncol Biol Phys. 2013;85:846–853. 22749634 10.1016/j.ijrobp.2012.05.026

[20] Boswell S , Tome W , Jeraj R , Jaradat H , Mackie TR . Automatic registration of megavoltage to kilovoltage CT images in helical tomotherapy: an evaluation of the setup verification process for the special case of a rigid head phantom. Med Phys. 2006;33:4395–4404. 17153418 10.1118/1.2349698

[21] Houghton F , Benson RJ , Tudor GS , et al An assessment of action levels in imaging strategies in head and neck cancer using TomoTherapy. Are our margins adequate in the absence of image guidance? Clin Oncol (R Coll Radiol). 2009;21:720–727. 19740637 10.1016/j.clon.2009.08.005

[22] Vaandering A , Lee JA , Renard L , Gregoire V . Evaluation of MVCT protocols for brain and head and neck tumor patients treated with helical tomotherapy. Radiother Oncol. 2009;93:50–56. 19515441 10.1016/j.radonc.2009.05.009

[23] Zumsteg Z , DeMarco J , Lee SP , et al Image guidance during head‐and‐neck cancer radiation therapy: analysis of alignment trends with in‐room cone‐beam computed tomography scans. Int J Radiat Oncol Biol Phys. 2012;83:712–719. 22099037 10.1016/j.ijrobp.2011.08.001

[24] Yu Y , Michaud AL , Sreeraman R , Liu T , Purdy JA , Chen AM . Comparison of daily versus nondaily image‐guided radiotherapy protocols for patients treated with intensity‐modulated radiotherapy for head and neck cancer. Head Neck. 2014;36:992–997. 23780718 10.1002/hed.23401

[25] Kim S , Akpati HC , Kielbasa JE , et al Evaluation of intrafraction patient movement for CNS and head & neck IMRT. Med Phys. 2004;31:500–506. 15070246 10.1118/1.1644641

[26] Gurney‐Champion OJ , McQuaid D , Dunlop A , et al MRI‐based Assessment of 3D Intrafractional Motion of Head and Neck Cancer for Radiation Therapy. Int J Radiat Oncol Biol Phys. 2018;100:306–316. 29229323 10.1016/j.ijrobp.2017.10.016 PMC5777665

[27] Pang PP , Hendry J , Cheah SL , et al An assessment of the magnitude of intra‐fraction movement of head‐and‐neck IMRT cases and its implication on the action‐level of the imaging protocol. Radiother Oncol. 2014;112:437–441. 25284062 10.1016/j.radonc.2014.09.008

[28] Suzuki M , Nishimura Y , Nakamatsu K , et al Analysis of interfractional set‐up errors and intrafractional organ motions during IMRT for head and neck tumors to define an appropriate planning target volume (PTV)‐ and planning organs at risk volume (PRV)‐margins. Radiother Oncol. 2006;78:283–290. 16564594 10.1016/j.radonc.2006.03.006

[29] Drabik DM , MacKenzie MA , Fallone GB . Quantifying appropriate PTV setup margins: analysis of patient setup fidelity and intrafraction motion using post‐treatment megavoltage computed tomography scans. Int J Radiat Oncol Biol Phys. 2007;68:1222–1228. 17637395 10.1016/j.ijrobp.2007.04.007

[30] Beltran C , Trussell J , Merchant TE . Dosimetric impact of intrafractional patient motion in pediatric brain tumor patients. Med Dosim. 2010;35:43–48. 19931014 10.1016/j.meddos.2009.01.004 PMC3800030

[31] Petersson K , Enmark M , Ceberg C , Knöös T . 1250 poster Increased patient throughput for treatment with helical tomotherapy. Radiother Oncol. 2011;99:S465–S466.

[32] Haraldsson A , Engellau J , Lenhoff S , Engelholm S , Bäck S , Engström PE . Implementing safe and robust total marrow irradiation using helical tomotherap ‐ a practical guide. Phys Med Eur J Med Phys. 2019;60:162–167. 10.1016/j.ejmp.2019.03.032 31000078

[33] Shah AP , Langen KM , Ruchala KJ , Cox A , Kupelian PA , Meeks SL . Patient dose from megavoltage computed tomography imaging. Int J Radiat Oncol Biol Phys. 2008;70:1579–1587. 18234438 10.1016/j.ijrobp.2007.11.048

